# (Ferrocenylmeth­yl)trimethyl­ammonium perchlorate

**DOI:** 10.1107/S1600536812002176

**Published:** 2012-01-21

**Authors:** Ying-Chun Wang

**Affiliations:** a College of Chemistry and Chemical Engineering, Southeast University, Nanjing 210096, People’s Republic of China

## Abstract

The asymmetric unit of the title complex, [Fe(C_5_H_5_)(C_9_H_15_N)]ClO_4_, contains one discrete (ferrocenylmeth­yl)trimethyl­ammonium cation and one perchlorate anion. The anion is disordered over two sets of sites, with refined occupancies of 0.776 (8) and 0.224 (8). The distances from the Fe atom to the centroids of the unsubstituted and substituted cyclo­penta­dienyl (Cp) rings are 1.650 (1) and 1.640 (1) Å, respectively. The Cp rings form a dihedral angle of 2.66 (3)°.

## Related literature

For a related structure, see: Pullen *et al.* (1998 ▶). For the ferroelectric properties of related amino derivatives, see: Fu *et al.* (2011*a* ▶,*b* ▶,*c* ▶); Fu *et al.* (2007 ▶, 2008 ▶, 2009 ▶); Fu & Xiong (2008 ▶).
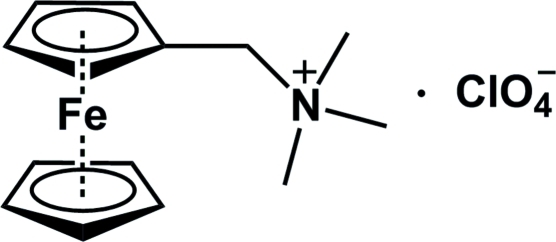


## Experimental

### 

#### Crystal data

[Fe(C_5_H_5_)(C_9_H_15_N)]ClO_4_
*M* *_r_* = 357.61
Monoclinic, 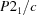

*a* = 8.5972 (17) Å
*b* = 13.783 (3) Å
*c* = 13.096 (3) Å
β = 101.23 (3)°
*V* = 1522.1 (6) Å^3^
*Z* = 4
Mo *K*α radiation
μ = 1.18 mm^−1^
*T* = 298 K
0.10 × 0.03 × 0.03 mm

#### Data collection

Rigaku Mercury2 diffractometer
Absorption correction: multi-scan (*CrystalClear*; Rigaku, 2005 ▶) *T*_min_ = 0.910, *T*_max_ = 1.000
15527 measured reflections
3479 independent reflections
2642 reflections with *I* > 2σ(*I*)
*R*_int_ = 0.062

#### Refinement

*R*[*F* ^2^ > 2σ(*F* ^2^)] = 0.057
*wR*(*F* ^2^) = 0.148
*S* = 1.07
3479 reflections
218 parameters
H-atom parameters constrained
Δρ_max_ = 0.32 e Å^−3^
Δρ_min_ = −0.73 e Å^−3^

### 

Data collection: *CrystalClear* (Rigaku, 2005 ▶); cell refinement: *CrystalClear*; data reduction: *CrystalClear*; program(s) used to solve structure: *SHELXS97* (Sheldrick, 2008 ▶); program(s) used to refine structure: *SHELXL97* (Sheldrick, 2008 ▶); molecular graphics: *SHELXTL* (Sheldrick, 2008 ▶); software used to prepare material for publication: *SHELXTL*.

## Supplementary Material

Crystal structure: contains datablock(s) I, global. DOI: 10.1107/S1600536812002176/lh5405sup1.cif

Structure factors: contains datablock(s) I. DOI: 10.1107/S1600536812002176/lh5405Isup2.hkl

Additional supplementary materials:  crystallographic information; 3D view; checkCIF report

## Figures and Tables

**Fig. 1.**
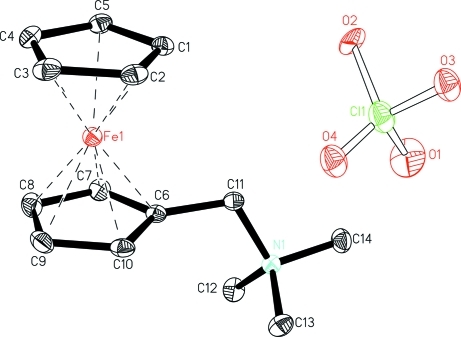
A view of the title compound with displacement ellipsoids drawn at the 30% probability level. The disorder is not shown.

## supplementary crystallographic information

### Comment

Simple organic salts containig amino cations have attracted an attention as materials which display ferroelectric-paraelectric phase transitions (Fu *et al.*, 2011*a*, *b* and *c*). With the purpose of obtaining phase transition crystals of amino compounds, various amines have been studied and a series of new materials with this organic molecules have been elaborated (Fu *et al.* 2007, 2008, 2009; Fu & Xiong 2008). Herein we present the crystal structure of the title compound (I), which may be used as a cation in organic salts. In this study, we describe the crystal structure of this compound.

The asymmetric unit of (I) contains one discrete trimethyl(ferrocenyl)methylammonium cation and one ClO_4_^-^ anion (Fig. 1). The anion is disordered over two sets of sites with refined occupancies 0.776 (8) and 0.224 (8). The distances from the Fe atom to the centroids of the unsubstituted and substituted cyclopentadienyl (Cp) rings are 1.650 (1) and 1.640 (1)Å , respectively. The dihedral angles between the two Cp rings are 2.66 (3)°. The two cyclopentadienyl rings of the ferrocenyl group are almost eclipsed with the (C—Cg1—Cg2—C) torsion angles in the two Cp rings in the range of 3.67 (3) to 4.74 (3)°. For a comparison of bond lengths and angles, see those in the related structure (Pullen *et al.*, 1998).

### Experimental

A mixture of commercial trimethyl(ferrocenyl)methylamine (0.4 mmol) and HClO_4_ (0.4 mmol) were dissolved in EtOH/distilled water (1:1 *v*/*v*) solvent. The solution was slowly evaporated in air affording red block-shaped crystals of the title compound suitable for X-ray analysis.

### Refinement

All H atoms attached to C atoms were fixed geometrically and treated as riding with C-H = 0.97 Å(C methylene), C-H = 0.98 Å(C ferrocenyl) and C-H = 0.96 Å(C methyl) with *U*_iso_(H) = 1.2*U*_eq_(C except methyl) and *U*_iso_(H) = 1.5*U*_eq_(methyl).

The ClO_4_^-^ anion is disordered over sites and refined using the PART in struction in SHELXL (Sheldrick, 2008).

### Crystal data

**Table:**

[Fe(C_5_H_5_)(C_9_H_15_N)]ClO_4_	*F*(000) = 744
*M**_r_* = 357.61	*D*_x_ = 1.561 Mg m^−^^3^
Monoclinic, *P*2_1_/*c*	Mo *K*α radiation, λ = 0.71073 Å
Hall symbol: -P 2ybc	Cell parameters from 3479 reflections
*a* = 8.5972 (17) Å	θ = 3.0–27.5°
*b* = 13.783 (3) Å	µ = 1.18 mm^−^^1^
*c* = 13.096 (3) Å	*T* = 298 K
β = 101.23 (3)°	Block, red
*V* = 1522.1 (6) Å^3^	0.10 × 0.03 × 0.03 mm
*Z* = 4	

### Data collection

**Table:**

Rigaku Mercury2 diffractometer	3479 independent reflections
Radiation source: fine-focus sealed tube	2642 reflections with *I* > 2σ(*I*)
graphite	*R*_int_ = 0.062
Detector resolution: 13.6612 pixels mm^-1^	θ_max_ = 27.5°, θ_min_ = 3.0°
CCD profile fitting scans	*h* = −11→11
Absorption correction: multi-scan (*CrystalClear*; Rigaku, 2005)	*k* = −17→17
*T*_min_ = 0.910, *T*_max_ = 1.000	*l* = −16→17
15527 measured reflections	

### Refinement

**Table:**

Refinement on *F*^2^	Primary atom site location: structure-invariant direct methods
Least-squares matrix: full	Secondary atom site location: difference Fourier map
*R*[*F*^2^ > 2σ(*F*^2^)] = 0.057	Hydrogen site location: inferred from neighbouring sites
*wR*(*F*^2^) = 0.148	H-atom parameters constrained
*S* = 1.07	*w* = 1/[σ^2^(*F*_o_^2^) + (0.0595*P*)^2^ + 2.0318*P*] where *P* = (*F*_o_^2^ + 2*F*_c_^2^)/3
3479 reflections	(Δ/σ)_max_ < 0.001
218 parameters	Δρ_max_ = 0.32 e Å^−^^3^
0 restraints	Δρ_min_ = −0.73 e Å^−^^3^

### Special details

**Table:**

Experimental. The dielectric constant of title compound as a function of temperature indicates that the permittivity is basically temperature-independent, suggesting that this compound should be not a real ferroelectrics or there may be no distinct phase transition occurred within the measured temperature range. Similarly, below the melting point (411 K) of the compound, the dielectric constant as a function of temperature also goes smoothly, and there is no dielectric anomaly observed (dielectric constant ranging from 4.4 to 9.5).
Geometry. All esds (except the esd in the dihedral angle between two l.s. planes) are estimated using the full covariance matrix. The cell esds are taken into account individually in the estimation of esds in distances, angles and torsion angles; correlations between esds in cell parameters are only used when they are defined by crystal symmetry. An approximate (isotropic) treatment of cell esds is used for estimating esds involving l.s. planes.
Refinement. Refinement of F^2^ against ALL reflections. The weighted R-factor wR and goodness of fit S are based on F^2^, conventional R-factors R are based on F, with F set to zero for negative F^2^. The threshold expression of F^2^ > 2sigma(F^2^) is used only for calculating R-factors(gt) etc. and is not relevant to the choice of reflections for refinement. R-factors based on F^2^ are statistically about twice as large as those based on F, and R- factors based on ALL data will be even larger.

### Fractional atomic coordinates and isotropic or equivalent isotropic displacement parameters (Å^2^)

**Table:**

	*x*	*y*	*z*	*U*_iso_*/*U*_eq_	Occ. (<1)
Fe1	0.66928 (6)	0.67531 (3)	0.46111 (4)	0.03474 (18)	
N1	0.2467 (4)	0.8607 (2)	0.3523 (2)	0.0387 (7)	
C1	0.6435 (5)	0.6059 (3)	0.3215 (3)	0.0439 (9)	
H1A	0.5675	0.6231	0.2581	0.053*	
C2	0.6154 (5)	0.5411 (3)	0.3991 (3)	0.0463 (9)	
H2A	0.5169	0.5052	0.3989	0.056*	
C3	0.7542 (6)	0.5370 (3)	0.4773 (4)	0.0528 (11)	
H3A	0.7694	0.4977	0.5408	0.063*	
C4	0.8680 (5)	0.5997 (3)	0.4467 (3)	0.0516 (10)	
H4A	0.9756	0.6117	0.4857	0.062*	
C5	0.7992 (5)	0.6413 (3)	0.3501 (3)	0.0469 (9)	
H5A	0.8503	0.6877	0.3105	0.056*	
C6	0.4993 (5)	0.7790 (2)	0.4457 (3)	0.0371 (8)	
C7	0.6527 (5)	0.8227 (3)	0.4722 (3)	0.0476 (10)	
H7A	0.6967	0.8702	0.4299	0.057*	
C8	0.7317 (6)	0.7840 (3)	0.5682 (4)	0.0568 (11)	
H8A	0.8396	0.7999	0.6041	0.068*	
C9	0.6286 (6)	0.7178 (3)	0.6028 (3)	0.0577 (12)	
H9A	0.6532	0.6796	0.6670	0.069*	
C10	0.4849 (5)	0.7132 (3)	0.5283 (3)	0.0455 (9)	
H10A	0.3925	0.6730	0.5327	0.055*	
C11	0.3848 (5)	0.7937 (3)	0.3457 (3)	0.0415 (8)	
H11A	0.3429	0.7310	0.3203	0.050*	
H11B	0.4419	0.8200	0.2949	0.050*	
C12	0.3026 (6)	0.9568 (3)	0.3968 (4)	0.0570 (11)	
H12A	0.3564	0.9487	0.4677	0.085*	
H12B	0.2133	0.9991	0.3944	0.085*	
H12C	0.3741	0.9845	0.3569	0.085*	
C13	0.1414 (5)	0.8167 (3)	0.4173 (4)	0.0587 (11)	
H13A	0.2010	0.8051	0.4862	0.088*	
H13B	0.1000	0.7563	0.3869	0.088*	
H13C	0.0554	0.8601	0.4208	0.088*	
C14	0.1527 (6)	0.8749 (4)	0.2435 (3)	0.0603 (12)	
H14A	0.0651	0.9177	0.2455	0.091*	
H14B	0.1134	0.8134	0.2152	0.091*	
H14C	0.2197	0.9028	0.2006	0.091*	
Cl2	0.18301 (16)	0.55643 (10)	0.19867 (10)	0.0700 (4)	0.776 (8)
O1'	0.1752 (13)	0.5939 (6)	0.2902 (6)	0.155 (4)	0.776 (8)
O2'	0.2997 (11)	0.6113 (8)	0.1573 (5)	0.126 (4)	0.776 (8)
O3'	0.0584 (9)	0.5454 (9)	0.1175 (7)	0.176 (5)	0.776 (8)
O4'	0.2388 (19)	0.4651 (7)	0.2210 (8)	0.226 (6)	0.776 (8)
Cl1	0.18301 (16)	0.55643 (10)	0.19867 (10)	0.0700 (4)	0.224 (8)
O1	0.109 (3)	0.6459 (15)	0.174 (2)	0.120 (10)	0.224 (8)
O2	0.318 (2)	0.547 (2)	0.155 (2)	0.100 (11)	0.224 (8)
O4	0.204 (2)	0.5450 (16)	0.2919 (16)	0.0700 (4)	0.224 (8)
O3	0.107 (3)	0.4943 (15)	0.1361 (16)	0.0700 (4)	0.224 (8)

### Atomic displacement parameters (Å^2^)

**Table:**

	*U*^11^	*U*^22^	*U*^33^	*U*^12^	*U*^13^	*U*^23^
Fe1	0.0385 (3)	0.0296 (3)	0.0361 (3)	0.0032 (2)	0.0074 (2)	0.0004 (2)
N1	0.0406 (17)	0.0310 (15)	0.0446 (17)	0.0015 (13)	0.0084 (14)	0.0006 (13)
C1	0.049 (2)	0.042 (2)	0.042 (2)	0.0069 (17)	0.0115 (17)	−0.0069 (16)
C2	0.050 (2)	0.0316 (19)	0.062 (3)	0.0002 (16)	0.022 (2)	−0.0085 (17)
C3	0.068 (3)	0.036 (2)	0.059 (3)	0.020 (2)	0.024 (2)	0.0103 (18)
C4	0.040 (2)	0.057 (3)	0.057 (2)	0.0133 (19)	0.0088 (19)	0.002 (2)
C5	0.043 (2)	0.052 (2)	0.051 (2)	0.0006 (18)	0.0206 (19)	0.0021 (19)
C6	0.045 (2)	0.0279 (18)	0.0401 (19)	0.0057 (15)	0.0118 (16)	−0.0019 (14)
C7	0.052 (2)	0.0315 (19)	0.059 (3)	−0.0043 (17)	0.008 (2)	−0.0072 (18)
C8	0.059 (3)	0.048 (2)	0.057 (3)	0.003 (2)	−0.006 (2)	−0.017 (2)
C9	0.080 (3)	0.057 (3)	0.035 (2)	0.021 (2)	0.008 (2)	−0.0040 (19)
C10	0.057 (2)	0.039 (2)	0.045 (2)	0.0123 (18)	0.0210 (19)	0.0012 (17)
C11	0.045 (2)	0.0354 (19)	0.045 (2)	0.0048 (16)	0.0113 (17)	−0.0035 (16)
C12	0.063 (3)	0.031 (2)	0.073 (3)	0.0022 (19)	0.003 (2)	−0.0063 (19)
C13	0.048 (2)	0.064 (3)	0.068 (3)	−0.003 (2)	0.020 (2)	0.005 (2)
C14	0.059 (3)	0.063 (3)	0.053 (3)	0.009 (2)	−0.003 (2)	0.002 (2)
Cl2	0.0726 (8)	0.0714 (9)	0.0628 (7)	−0.0219 (6)	0.0057 (6)	−0.0068 (6)
O1'	0.283 (11)	0.088 (5)	0.131 (6)	−0.044 (6)	0.131 (7)	−0.052 (5)
O2'	0.124 (7)	0.192 (9)	0.060 (4)	−0.107 (7)	0.012 (4)	0.007 (5)
O3'	0.089 (5)	0.237 (12)	0.166 (8)	−0.068 (6)	−0.063 (5)	0.090 (7)
O4'	0.406 (18)	0.094 (6)	0.182 (9)	0.117 (9)	0.066 (10)	0.035 (6)
Cl1	0.0726 (8)	0.0714 (9)	0.0628 (7)	−0.0219 (6)	0.0057 (6)	−0.0068 (6)
O1	0.125 (19)	0.056 (11)	0.16 (2)	0.058 (12)	−0.015 (16)	−0.007 (13)
O2	0.017 (7)	0.15 (2)	0.14 (2)	−0.028 (11)	0.024 (9)	−0.07 (2)
O4	0.0726 (8)	0.0714 (9)	0.0628 (7)	−0.0219 (6)	0.0057 (6)	−0.0068 (6)
O3	0.0726 (8)	0.0714 (9)	0.0628 (7)	−0.0219 (6)	0.0057 (6)	−0.0068 (6)

### Geometric parameters (Å, °)

**Table:**

Fe1—C10	2.025 (4)	C6—C10	1.435 (5)
Fe1—C6	2.026 (4)	C6—C11	1.491 (5)
Fe1—C1	2.037 (4)	C7—C8	1.412 (6)
Fe1—C2	2.037 (4)	C7—H7A	0.9800
Fe1—C3	2.037 (4)	C8—C9	1.407 (7)
Fe1—C9	2.040 (4)	C8—H8A	0.9800
Fe1—C4	2.041 (4)	C9—C10	1.418 (6)
Fe1—C7	2.044 (4)	C9—H9A	0.9800
Fe1—C8	2.051 (4)	C10—H10A	0.9800
Fe1—C5	2.053 (4)	C11—H11A	0.9700
N1—C13	1.488 (5)	C11—H11B	0.9700
N1—C12	1.489 (5)	C12—H12A	0.9600
N1—C14	1.507 (5)	C12—H12B	0.9600
N1—C11	1.520 (5)	C12—H12C	0.9600
C1—C5	1.405 (6)	C13—H13A	0.9600
C1—C2	1.408 (6)	C13—H13B	0.9600
C1—H1A	0.9800	C13—H13C	0.9600
C2—C3	1.415 (6)	C14—H14A	0.9600
C2—H2A	0.9800	C14—H14B	0.9600
C3—C4	1.420 (6)	C14—H14C	0.9600
C3—H3A	0.9800	Cl2—O1'	1.319 (6)
C4—C5	1.410 (6)	Cl2—O4'	1.358 (7)
C4—H4A	0.9800	Cl2—O3'	1.363 (7)
C5—H5A	0.9800	Cl2—O2'	1.443 (6)
C6—C7	1.430 (5)		
			
C10—Fe1—C6	41.48 (15)	C3—C4—Fe1	69.5 (2)
C10—Fe1—C1	123.60 (17)	C5—C4—H4A	126.0
C6—Fe1—C1	106.95 (16)	C3—C4—H4A	126.0
C10—Fe1—C2	105.73 (17)	Fe1—C4—H4A	126.0
C6—Fe1—C2	119.82 (16)	C1—C5—C4	108.0 (4)
C1—Fe1—C2	40.45 (16)	C1—C5—Fe1	69.3 (2)
C10—Fe1—C3	119.43 (17)	C4—C5—Fe1	69.4 (2)
C6—Fe1—C3	155.14 (18)	C1—C5—H5A	126.0
C1—Fe1—C3	68.17 (17)	C4—C5—H5A	126.0
C2—Fe1—C3	40.63 (18)	Fe1—C5—H5A	126.0
C10—Fe1—C9	40.84 (18)	C7—C6—C10	107.1 (4)
C6—Fe1—C9	68.88 (16)	C7—C6—C11	125.1 (3)
C1—Fe1—C9	160.6 (2)	C10—C6—C11	127.5 (4)
C2—Fe1—C9	123.89 (19)	C7—C6—Fe1	70.1 (2)
C3—Fe1—C9	107.20 (18)	C10—C6—Fe1	69.2 (2)
C10—Fe1—C4	155.77 (17)	C11—C6—Fe1	121.6 (2)
C6—Fe1—C4	162.09 (17)	C8—C7—C6	108.5 (4)
C1—Fe1—C4	67.90 (17)	C8—C7—Fe1	70.1 (2)
C2—Fe1—C4	68.21 (17)	C6—C7—Fe1	68.8 (2)
C3—Fe1—C4	40.75 (18)	C8—C7—H7A	125.7
C9—Fe1—C4	121.73 (18)	C6—C7—H7A	125.7
C10—Fe1—C7	68.97 (17)	Fe1—C7—H7A	125.7
C6—Fe1—C7	41.13 (15)	C9—C8—C7	107.9 (4)
C1—Fe1—C7	122.26 (17)	C9—C8—Fe1	69.5 (2)
C2—Fe1—C7	156.55 (17)	C7—C8—Fe1	69.6 (2)
C3—Fe1—C7	162.02 (18)	C9—C8—H8A	126.0
C9—Fe1—C7	67.86 (18)	C7—C8—H8A	126.0
C4—Fe1—C7	125.84 (18)	Fe1—C8—H8A	126.0
C10—Fe1—C8	68.72 (19)	C8—C9—C10	109.0 (4)
C6—Fe1—C8	68.92 (17)	C8—C9—Fe1	70.3 (3)
C1—Fe1—C8	157.69 (19)	C10—C9—Fe1	69.0 (2)
C2—Fe1—C8	160.89 (19)	C8—C9—H9A	125.5
C3—Fe1—C8	124.85 (19)	C10—C9—H9A	125.5
C9—Fe1—C8	40.2 (2)	Fe1—C9—H9A	125.5
C4—Fe1—C8	108.92 (19)	C9—C10—C6	107.4 (4)
C7—Fe1—C8	40.35 (17)	C9—C10—Fe1	70.1 (2)
C10—Fe1—C5	161.13 (17)	C6—C10—Fe1	69.3 (2)
C6—Fe1—C5	124.89 (16)	C9—C10—H10A	126.3
C1—Fe1—C5	40.19 (16)	C6—C10—H10A	126.3
C2—Fe1—C5	67.84 (17)	Fe1—C10—H10A	126.3
C3—Fe1—C5	68.07 (17)	C6—C11—N1	115.0 (3)
C9—Fe1—C5	157.41 (19)	C6—C11—H11A	108.5
C4—Fe1—C5	40.28 (17)	N1—C11—H11A	108.5
C7—Fe1—C5	109.43 (18)	C6—C11—H11B	108.5
C8—Fe1—C5	123.05 (19)	N1—C11—H11B	108.5
C13—N1—C12	108.9 (3)	H11A—C11—H11B	107.5
C13—N1—C14	108.7 (3)	N1—C12—H12A	109.5
C12—N1—C14	109.1 (3)	N1—C12—H12B	109.5
C13—N1—C11	110.7 (3)	H12A—C12—H12B	109.5
C12—N1—C11	111.5 (3)	N1—C12—H12C	109.5
C14—N1—C11	107.9 (3)	H12A—C12—H12C	109.5
C5—C1—C2	108.4 (4)	H12B—C12—H12C	109.5
C5—C1—Fe1	70.5 (2)	N1—C13—H13A	109.5
C2—C1—Fe1	69.8 (2)	N1—C13—H13B	109.5
C5—C1—H1A	125.8	H13A—C13—H13B	109.5
C2—C1—H1A	125.8	N1—C13—H13C	109.5
Fe1—C1—H1A	125.8	H13A—C13—H13C	109.5
C1—C2—C3	108.0 (4)	H13B—C13—H13C	109.5
C1—C2—Fe1	69.7 (2)	N1—C14—H14A	109.5
C3—C2—Fe1	69.7 (2)	N1—C14—H14B	109.5
C1—C2—H2A	126.0	H14A—C14—H14B	109.5
C3—C2—H2A	126.0	N1—C14—H14C	109.5
Fe1—C2—H2A	126.0	H14A—C14—H14C	109.5
C2—C3—C4	107.6 (4)	H14B—C14—H14C	109.5
C2—C3—Fe1	69.7 (2)	O1'—Cl2—O4'	104.2 (6)
C4—C3—Fe1	69.8 (2)	O1'—Cl2—O3'	125.5 (8)
C2—C3—H3A	126.2	O4'—Cl2—O3'	104.8 (8)
C4—C3—H3A	126.2	O1'—Cl2—O2'	107.3 (5)
Fe1—C3—H3A	126.2	O4'—Cl2—O2'	108.9 (8)
C5—C4—C3	108.0 (4)	O3'—Cl2—O2'	105.3 (5)
C5—C4—Fe1	70.3 (2)		
			
C10—Fe1—C1—C5	166.9 (2)	C3—Fe1—C6—C10	47.4 (5)
C6—Fe1—C1—C5	124.5 (2)	C9—Fe1—C6—C10	−38.0 (3)
C2—Fe1—C1—C5	−119.2 (4)	C4—Fe1—C6—C10	−167.7 (5)
C3—Fe1—C1—C5	−81.4 (3)	C7—Fe1—C6—C10	−118.1 (3)
C9—Fe1—C1—C5	−161.1 (5)	C8—Fe1—C6—C10	−81.3 (3)
C4—Fe1—C1—C5	−37.3 (3)	C5—Fe1—C6—C10	162.4 (2)
C7—Fe1—C1—C5	82.1 (3)	C10—Fe1—C6—C11	−122.1 (4)
C8—Fe1—C1—C5	48.8 (5)	C1—Fe1—C6—C11	−0.2 (3)
C10—Fe1—C1—C2	−73.9 (3)	C2—Fe1—C6—C11	−42.3 (4)
C6—Fe1—C1—C2	−116.3 (2)	C3—Fe1—C6—C11	−74.7 (5)
C3—Fe1—C1—C2	37.8 (3)	C9—Fe1—C6—C11	−160.2 (4)
C9—Fe1—C1—C2	−41.9 (6)	C4—Fe1—C6—C11	70.2 (6)
C4—Fe1—C1—C2	81.9 (3)	C7—Fe1—C6—C11	119.8 (4)
C7—Fe1—C1—C2	−158.7 (2)	C8—Fe1—C6—C11	156.6 (4)
C8—Fe1—C1—C2	168.0 (4)	C5—Fe1—C6—C11	40.2 (4)
C5—Fe1—C1—C2	119.2 (4)	C10—C6—C7—C8	0.7 (4)
C5—C1—C2—C3	0.7 (4)	C11—C6—C7—C8	−174.1 (4)
Fe1—C1—C2—C3	−59.4 (3)	Fe1—C6—C7—C8	−58.9 (3)
C5—C1—C2—Fe1	60.2 (3)	C10—C6—C7—Fe1	59.6 (2)
C10—Fe1—C2—C1	123.8 (2)	C11—C6—C7—Fe1	−115.2 (3)
C6—Fe1—C2—C1	81.1 (3)	C10—Fe1—C7—C8	81.5 (3)
C3—Fe1—C2—C1	−119.1 (4)	C6—Fe1—C7—C8	120.2 (4)
C9—Fe1—C2—C1	164.5 (2)	C1—Fe1—C7—C8	−161.2 (3)
C4—Fe1—C2—C1	−81.0 (3)	C2—Fe1—C7—C8	162.5 (4)
C7—Fe1—C2—C1	50.5 (5)	C3—Fe1—C7—C8	−39.9 (7)
C8—Fe1—C2—C1	−166.0 (5)	C9—Fe1—C7—C8	37.4 (3)
C5—Fe1—C2—C1	−37.5 (2)	C4—Fe1—C7—C8	−76.6 (3)
C10—Fe1—C2—C3	−117.1 (3)	C5—Fe1—C7—C8	−118.6 (3)
C6—Fe1—C2—C3	−159.7 (2)	C10—Fe1—C7—C6	−38.7 (2)
C1—Fe1—C2—C3	119.1 (4)	C1—Fe1—C7—C6	78.5 (3)
C9—Fe1—C2—C3	−76.4 (3)	C2—Fe1—C7—C6	42.3 (5)
C4—Fe1—C2—C3	38.1 (3)	C3—Fe1—C7—C6	−160.1 (5)
C7—Fe1—C2—C3	169.6 (4)	C9—Fe1—C7—C6	−82.8 (3)
C8—Fe1—C2—C3	−46.9 (6)	C4—Fe1—C7—C6	163.2 (2)
C5—Fe1—C2—C3	81.7 (3)	C8—Fe1—C7—C6	−120.2 (4)
C1—C2—C3—C4	−0.3 (4)	C5—Fe1—C7—C6	121.2 (2)
Fe1—C2—C3—C4	−59.7 (3)	C6—C7—C8—C9	−0.9 (5)
C1—C2—C3—Fe1	59.5 (3)	Fe1—C7—C8—C9	−59.1 (3)
C10—Fe1—C3—C2	79.7 (3)	C6—C7—C8—Fe1	58.1 (3)
C6—Fe1—C3—C2	45.6 (5)	C10—Fe1—C8—C9	37.2 (3)
C1—Fe1—C3—C2	−37.6 (2)	C6—Fe1—C8—C9	81.8 (3)
C9—Fe1—C3—C2	122.4 (3)	C1—Fe1—C8—C9	165.2 (4)
C4—Fe1—C3—C2	−118.6 (4)	C2—Fe1—C8—C9	−39.2 (7)
C7—Fe1—C3—C2	−166.6 (5)	C3—Fe1—C8—C9	−74.6 (3)
C8—Fe1—C3—C2	163.1 (3)	C4—Fe1—C8—C9	−117.1 (3)
C5—Fe1—C3—C2	−81.1 (3)	C7—Fe1—C8—C9	119.3 (4)
C10—Fe1—C3—C4	−161.7 (3)	C5—Fe1—C8—C9	−159.5 (3)
C6—Fe1—C3—C4	164.3 (3)	C10—Fe1—C8—C7	−82.2 (3)
C1—Fe1—C3—C4	81.0 (3)	C6—Fe1—C8—C7	−37.5 (2)
C2—Fe1—C3—C4	118.6 (4)	C1—Fe1—C8—C7	45.8 (6)
C9—Fe1—C3—C4	−119.0 (3)	C2—Fe1—C8—C7	−158.6 (5)
C7—Fe1—C3—C4	−47.9 (7)	C3—Fe1—C8—C7	166.1 (3)
C8—Fe1—C3—C4	−78.3 (3)	C9—Fe1—C8—C7	−119.3 (4)
C5—Fe1—C3—C4	37.6 (3)	C4—Fe1—C8—C7	123.5 (3)
C2—C3—C4—C5	−0.3 (5)	C5—Fe1—C8—C7	81.2 (3)
Fe1—C3—C4—C5	−60.0 (3)	C7—C8—C9—C10	0.9 (5)
C2—C3—C4—Fe1	59.7 (3)	Fe1—C8—C9—C10	−58.3 (3)
C10—Fe1—C4—C5	160.8 (4)	C7—C8—C9—Fe1	59.2 (3)
C6—Fe1—C4—C5	−39.3 (6)	C10—Fe1—C9—C8	−120.5 (4)
C1—Fe1—C4—C5	37.2 (3)	C6—Fe1—C9—C8	−81.9 (3)
C2—Fe1—C4—C5	81.0 (3)	C1—Fe1—C9—C8	−163.0 (5)
C3—Fe1—C4—C5	119.0 (4)	C2—Fe1—C9—C8	165.6 (3)
C9—Fe1—C4—C5	−161.7 (3)	C3—Fe1—C9—C8	124.1 (3)
C7—Fe1—C4—C5	−77.4 (3)	C4—Fe1—C9—C8	81.9 (3)
C8—Fe1—C4—C5	−119.2 (3)	C7—Fe1—C9—C8	−37.5 (3)
C10—Fe1—C4—C3	41.8 (6)	C5—Fe1—C9—C8	50.0 (6)
C6—Fe1—C4—C3	−158.3 (5)	C6—Fe1—C9—C10	38.6 (2)
C1—Fe1—C4—C3	−81.7 (3)	C1—Fe1—C9—C10	−42.4 (6)
C2—Fe1—C4—C3	−38.0 (3)	C2—Fe1—C9—C10	−73.9 (3)
C9—Fe1—C4—C3	79.3 (3)	C3—Fe1—C9—C10	−115.4 (3)
C7—Fe1—C4—C3	163.6 (3)	C4—Fe1—C9—C10	−157.5 (3)
C8—Fe1—C4—C3	121.9 (3)	C7—Fe1—C9—C10	83.0 (3)
C5—Fe1—C4—C3	−119.0 (4)	C8—Fe1—C9—C10	120.5 (4)
C2—C1—C5—C4	−0.9 (5)	C5—Fe1—C9—C10	170.5 (4)
Fe1—C1—C5—C4	58.8 (3)	C8—C9—C10—C6	−0.4 (5)
C2—C1—C5—Fe1	−59.7 (3)	Fe1—C9—C10—C6	−59.5 (3)
C3—C4—C5—C1	0.8 (5)	C8—C9—C10—Fe1	59.1 (3)
Fe1—C4—C5—C1	−58.7 (3)	C7—C6—C10—C9	−0.1 (4)
C3—C4—C5—Fe1	59.5 (3)	C11—C6—C10—C9	174.5 (3)
C10—Fe1—C5—C1	−35.7 (6)	Fe1—C6—C10—C9	60.0 (3)
C6—Fe1—C5—C1	−74.1 (3)	C7—C6—C10—Fe1	−60.2 (3)
C2—Fe1—C5—C1	37.7 (2)	C11—C6—C10—Fe1	114.5 (4)
C3—Fe1—C5—C1	81.7 (3)	C6—Fe1—C10—C9	−118.5 (4)
C9—Fe1—C5—C1	163.7 (4)	C1—Fe1—C10—C9	164.4 (3)
C4—Fe1—C5—C1	119.7 (4)	C2—Fe1—C10—C9	124.0 (3)
C7—Fe1—C5—C1	−117.4 (3)	C3—Fe1—C10—C9	82.3 (3)
C8—Fe1—C5—C1	−160.1 (2)	C4—Fe1—C10—C9	52.3 (5)
C10—Fe1—C5—C4	−155.3 (5)	C7—Fe1—C10—C9	−80.1 (3)
C6—Fe1—C5—C4	166.3 (2)	C8—Fe1—C10—C9	−36.7 (3)
C1—Fe1—C5—C4	−119.7 (4)	C5—Fe1—C10—C9	−168.7 (5)
C2—Fe1—C5—C4	−82.0 (3)	C1—Fe1—C10—C6	−77.1 (3)
C3—Fe1—C5—C4	−38.0 (3)	C2—Fe1—C10—C6	−117.5 (2)
C9—Fe1—C5—C4	44.1 (6)	C3—Fe1—C10—C6	−159.2 (2)
C7—Fe1—C5—C4	123.0 (3)	C9—Fe1—C10—C6	118.5 (4)
C8—Fe1—C5—C4	80.2 (3)	C4—Fe1—C10—C6	170.8 (4)
C10—Fe1—C6—C7	118.1 (3)	C7—Fe1—C10—C6	38.4 (2)
C1—Fe1—C6—C7	−120.0 (2)	C8—Fe1—C10—C6	81.8 (3)
C2—Fe1—C6—C7	−162.0 (2)	C5—Fe1—C10—C6	−50.3 (6)
C3—Fe1—C6—C7	165.5 (4)	C7—C6—C11—N1	−104.2 (4)
C9—Fe1—C6—C7	80.1 (3)	C10—C6—C11—N1	82.1 (5)
C4—Fe1—C6—C7	−49.6 (6)	Fe1—C6—C11—N1	169.0 (2)
C8—Fe1—C6—C7	36.8 (3)	C13—N1—C11—C6	−66.6 (4)
C5—Fe1—C6—C7	−79.5 (3)	C12—N1—C11—C6	54.9 (4)
C1—Fe1—C6—C10	121.9 (3)	C14—N1—C11—C6	174.6 (3)
C2—Fe1—C6—C10	79.8 (3)		
